# Effects of Dementia Simulation on Nursing Student's Knowledge and Empathy

**DOI:** 10.1093/geroni/igaa057.005

**Published:** 2020-12-16

**Authors:** Michelle Kimzey, Jodi Patterson

**Affiliations:** Texas Christian University, Fort Worth, Texas, United States

## Abstract

The numbers of people living with dementia are overwhelming. Dementia education is important to prepare nursing students to care for this population. Care for people with dementia requires a better understanding of the reality of dementia. Dementia education must empower nursing students to appreciate, provide care, and support the needs of the caregiver and people with dementia. The purpose of this study was (1) to determine the effects of the dementia simulation on nursing students’ knowledge of dementia and empathy for people living with dementia, (2) to explore the level of empathic concern, shared affect, perspective-taking self and perspective-taking other of undergraduate nursing students towards individuals with dementia. A convergent mixed method design was used with pretest and posttest serving as the quantitative arm of the study. A focus group discussion with themes extracted served as the qualitative piece of the study. The convenience sample of 65 undergraduate nursing students. Students completed pre and post-test surveys which included Dementia Knowledge Assessment Tool Version 2, Comprehensive State Empathy Scale, and demographic questionnaire. Content analysis was conducted on focus group responses to qualitative interview questions. Quantitative results reported a significant increase in empathy. Qualitative findings supported the quantitative results. Results revealed the dementia simulation positively impacted students’ empathy for people with dementia. This learning activity was innovative and created excitement and intrigue about caring for people with dementia. The dementia simulation experience created an awareness of dementia and ignited nursing students’ passion to care for this population.

